# Anomalies of Imagination, Self-Disorders, and Schizophrenia Spectrum Psychopathology: A Network Analysis

**DOI:** 10.3389/fpsyt.2021.808009

**Published:** 2022-01-17

**Authors:** Andreas Rosén Rasmussen, Andrea Raballo, Antonio Preti, Ditte Sæbye, Josef Parnas

**Affiliations:** ^1^Mental Health Center Amager, University of Copenhagen, Copenhagen, Denmark; ^2^Mental Health Center Glostrup, University of Copenhagen, Broendby, Denmark; ^3^Department of Clinical Medicine, University of Copenhagen, Copenhagen, Denmark; ^4^Division of Psychiatry, Clinical Psychology and Rehabilitation, Department of Medicine, University of Perugia, Perugia, Italy; ^5^Center for Translational, Phenomenological and Developmental Psychopathology (CTPDP), Perugia University Hospital, Perugia, Italy; ^6^Department of Neuroscience, University of Turin, Turin, Italy; ^7^Center for Clinical Research and Prevention, Bispebjerg and Frederiksberg Hospital, Copenhagen, Denmark; ^8^Center for Subjectivity Research, University of Copenhagen, Copenhagen, Denmark

**Keywords:** psychosis, EAFI, EASE, subjective experience, phenomenology

## Abstract

**Background:**

Anomalies of imagination encompass disturbances of the basic experiential structure of fantasies and imagery that can be explored in a semi-structured way with the Examination of Anomalous Fantasy and Imagination (EAFI). We aimed (1) to examine the distribution of anomalies of imagination among different diagnostic groups and a group of healthy controls, and (2) to examine their relation with disorders of basic self, perceptual disturbances and canonical state psychopathology of the schizophrenia-spectrum (positive, negative and general symptoms).

**Methods:**

The 81 participants included patients with schizophrenia or other non-affective psychosis (*N* = 32), schizotypal personality disorder (*N* = 15) or other mental illness (*N* = 16) and healthy controls (*N* = 18). The assessment encompassed EAFI, Examination of Anomalous Self-Experience (EASE), parts of Bonn Scale for the Assessment of Basic Symptoms (BSABS) and Positive and Negative Syndrome Scale (PANSS). For network analysis, the associations of EAFI with the other psychopathological variables were tested by Pearson's correlation coefficient and graphically represented using multidimensional clustering. Comparisons between correlations in the network were tested with Steiger's test.

**Results:**

Anomalies of imagination aggregated significantly in schizophrenia-spectrum disorders compared to other mental illness and healthy controls with no difference between schizophrenia and schizotypal disorder. In the network analysis, anomalies of imagination were closely inter-connected with self-disorders. Although, the anomalies of imagination correlated moderately with perceptual disturbance and positive, negative and general state symptomatology, these dimensions aggregated separately and relatively distant in the network.

**Conclusions:**

The results support that anomalies of imagination are highly characteristic of schizophrenia-spectrum disorders and closely related to self-disorders.

## Introduction

Patients often report alterations of imaginative experience such as the formation of mental images with vivid character, disturbing contents, and/or strong affects in terms of “fantasies,” “vivid images” or “daydreams.” However, mainstream psychopathology lacks suitable descriptors for this domain of psychopathology and in current clinical practice the rich variety of these complaints is only partly captured by notions such as obsession and rumination (1). Based on explorative, clinical-phenomenological interviews, we have previously suggested (2) that this area is highly relevant to differential diagnosis, especially in young, first-contact patients without easily recognizable psychotic presentation, that is, in detecting individuals with or in risk of psychosis.

Research investigating mental imagery in mental disorders has almost exclusively drawn on the computational theory of mental imagery (3). In this approach, which derives from experimental psychology, mental imagery is defined as an internal representation that codes information in the form of depiction and is assessed with experimental tasks (4). Regarding schizophrenia, two studies employing self-rating scales have reported a greater vividness of imagery within the schizophrenia-spectrum compared to healthy controls (5, 6), whereas a third study provided contrary findings regarding visual imagery (7). In our view, a detailed clinical description of variants of anomalous imaginative experience is still warranted and requires a conceptual approach tailored to the assessment of subjective experience.

This empirical study is based on a conceptual framework derived from classic phenomenology and contemporary philosophy of mind (8–10), where, briefly stated, imagination (aka fantasy) is understood as a sensory-like experience of an object as absent i.e., contrary to a perception, the object is tacitly experienced as not being present to the senses but entertained as a possibility or non-existent. Accordingly, the experiences addressed here take place in a clear consciousness, they are not located to the perceptual surroundings, and the patient is aware that the experience is a fantasy. Our research was originally motivated by reports from patients examined in the research on self-disorders carried out by our research group over the last two decades (11). Although, certain obsessive-ruminative experiences are included in the Examination of Anomalous Self-experience (EASE) (12), the domain of imagination was not specifically elaborated in the EASE or in other existing instruments addressing anomalous subjective experiences (13–15). Thus, it became apparent that this area required further phenomenological and conceptual clarification. Subsequently, we have over a period of several years explored salient features of disturbed imagination in a variety of psychiatric patients (1, 2, 16) and have developed an instrument, the Examination of Anomalous Fantasy and Imagination (EAFI) (17), assessing these experiential anomalies in a semi-structured, phenomenologically-oriented interview (see Supplementary Table 1, for a brief overview of the items).

The anomalies of imagination, which the EAFI explores, do not encompass all varieties of psychopathology related to imagination but focuses on basic disturbances of the experiential structure of imagination, which differ radically from the general experience of imagination as understood in canonical phenomenological accounts (8, 9). Indeed, ordinary imaginative experience typically involves a dissipating and vague sensory-like content experienced as inherently non-spatial and radically different from perceived pictures. In other words, mental imagery is normally not experienced as an introspectively stable entity, liable to inspection (10). However, we found that many patients, describe an articulated spatio-temporal structure of fantasy life [termed spatialization of imagination (1)]. That is, mental images endure for many seconds or minutes with stable positions and mutual relations of the elements, frequently eliciting an active exploration of the fantasy. The image may have an autonomous development, independently of the will of the subject (“like a movie”), and there may be a profound experiential distance between the experience of imagery and the sense of self (“I observe what happens in my head”). Besides spatialization of imagination, other clinically significant anomalies include a predominance of fantasy life, various idiosyncratic existential contents and subtle “as-if” disturbances of the tacit discrimination of fantasy from other experiential modalities such as memory (1, 2, 16). A broad range of ideations (such as “daydreams,” “fears,” anticipations, intrusions, paranoid or suicidal ideation) can involve such structural disturbances of imaginative experience. The following clinical vignette illustrates some of these features [see also (17) for clinical examples].

Anna, 22-year old university student diagnosed with schizotypal personality disorder, described that she often has sequences, like an inner movie, in the head. They are triggered unexpectedly. For example, if her mother has visited her at the ward, and she notices some dangerous looking characters outside. Then some inner images appear, where these people follow her mother and do all kinds of unspeakable things. The images are very detailed, everything has a clear location and the action develops for minutes on its own accord—as if it really happened. She is terrified, her heart racing, but also feels rage and can imagine all sort of ways these men get payback. She often tries to focus on something else or repress the images but never successfully. It feels as if these sequences are localized to the frontal lobes behind the forehead. She is always aware that it is a fantasy but adds that such things could actually happen.

We have previously hypothesized that these anomalies of imagination, assessed with the EAFI, are associated with schizophrenia-spectrum disorders and closely related to disorder of basic self (2). A large body of research has over the last decades corroborated this notion of disordered self as a core vulnerability phenotype of schizophrenia-spectrum disorders [for recent reviews see (11, 18)]. Self-disorders are non-psychotic, subjective anomalies. These disturbances affect the basic experiential self, which is at the root of existing as a self-present, demarcated and temporally stable subject of experience and action. This “core” or “minimal” self refers to the first-personal articulation of all experience (thoughts, images, perceptions etc.), also called “mineness,” “for-me-ness” or “ipseity” (ipse being latin for self, itself) (19, 20). For example, patients describe that thoughts appear anonymous or lacking mineness, the body or some of its parts are experienced as strange, alien or lifeless, perhaps with a sense of distance or disconnection between the mind and the body, or their sense of selfhood is ephemeral “as if he was a thing, a refrigerator, and not a human subject” (21). Patients may experience a deficient sense of the privacy of the inner world and various solipsistic experiences, such as a fleeting sense of being at the center of the world or that one's experiential field is the only extant reality (22). In clinical work and research, self-disorders can be reliably (23) explored with the Examination of Anomalous Self-Experience (EASE) (12). Empirical studies from different groups have shown a selective aggregation of these experiences in schizophrenia-spectrum disorders in clinical (24–29) as well as familial high-risk populations (30, 31). Indeed, such selective distribution within the schizophrenia spectrum has also been recently corroborated at meta-analytic level (32). Self-disorders are longitudinally associated with development of psychosis in patients (33) and help-seeking adolescents (34) and are associated with the longitudinal unfolding of other psychopathological dimensions, particularly negative symptoms (35). Furthermore, self-disorders possess trait-like characteristics, indeed, recent studies have demonstrated stability both in degree and patterns of self-disorders across periods of five years (36–38).

In this study we wish to address the following aims and hypotheses:

1) To examine the distribution of anomalies of imagination assessed with the EAFI among different diagnostic groups, with special focus on the schizophrenia-spectrum, and a control group of healthy students. We hypothesized that anomalies of imagination would aggregate in schizophrenia-spectrum disorders. Parts of these data have previously been published in a research letter (39).
2) To cross-sectionally examine the relation of anomalies of imagination to disorders of basic self, perceptual disturbances and canonical state psychopathology of the schizophrenia spectrum (positive, negative and general symptoms). For this purpose, we employed network analysis. We hypothesized that anomalies of imagination would be closely related to self-disorders and more peripherally related to perceptual disturbances and positive, negative and general symptoms.

## Materials and Methods

### Sample

All patients were referred to the study by clinicians from out- and inpatient facilities at the Mental Health Services in the Capital Region of Denmark, Copenhagen University Hospital between February 2016 and February 2017. The inclusion criteria were a clinical ICD-10 diagnosis within the schizophrenia-spectrum (schizophrenia, other non-affective psychosis and schizotypal disorder) or a clinical diagnosis of obsessive-compulsive disorder (OCD). OCD patients were chosen as a clinical control group, unrelated to the schizophrenia-spectrum, because obsessions involve imaginative experience (1). The patients had to be considered capable of participating in lengthy interviews. This naturally excluded agitated and severely psychotic patients. Additional exclusion criteria were primary or clinically dominating alcohol or substance abuse, organic brain disorder, mental retardation and involuntary admission or legal status. With the purpose of recruiting a healthy control group, we contacted all students in mandatory training rotation at Mental Health Center Glostrup. Thirty medical, nursing and nursing-assistant students were interviewed. However, only 18 students were included because 12 students fulfilled diagnostic criteria of a DSM diagnosis on a lifetime basis. This occurrence of lifetime psychopathology in a non-clinical population is in accord with previous family studies (40, 41) and register studies (42). All individuals participated upon written consent. The study was approved by the Danish Data Protection Agency and adhered to the ethical principles laid down by the Helsinki Declaration. According to Danish legislation, approval from The Danish National Committee on Health Research Ethics is not required for interview studies of this kind.

The total sample consists of 63 patients and 18 healthy students (*N* = 81). The patients received the following DSM-5 research diagnoses: schizophrenia (*N* = 20), non-affective psychosis (*N* = 12), schizotypal personality disorder (*N* = 15), OCD (*N* = 12), and major depression (*N* = 4).

### Assessment

The interviews were conducted in a semi-structured, conversational way following phenomenological principles (43–45) with a total duration of 2–6 h, often split into several sessions. Rating an item was never based on a simple “yes” or “no” answer but required examples described by the patients. All interviews were conducted by ARR, an experienced research clinician and first-author of the Examination of Anomalous Fantasy and Imagination (EAFI). Eighty percent of the patients gave consent to videotaping the interviews. After each interview, the interviewer made a detailed narrative summary of all sections of the interview schedule.

All patients were assessed for general psychopathology with a composite interview schedule used in several studies in our group (24, 37, 46, 47) consisting of the following elements: A thorough psychosocial history, a description of the illness evolution, the Operational Criteria Checklist (OPCRIT) (48) [created on the basis of Present State Examination (49)] expanded with additional items from the Schedule for Affective Disorders and Schizophrenia (SADS-L) (50) and a mental state examination targeting expressive features (e.g., affect modulation, stereotypies, mannerisms and formal thought disorder) (40, 51). State levels of positive, negative and general symptoms were assessed with the Positive and Negative Syndrome Scale (PANSS) (52). The split version of the Global Assessment of Functioning scale (GAF) (53) was also rated.

Anomalies of imagination were assessed with the EAFI (17) on a lifetime basis. The instrument encompasses 16 items (see Supplementary Table 1), some further divided into subtypes. Each item contains a definition of the experience being assessed (with considerations about the item's delimitation from other complaints) and prototypical examples of the patients' self-descriptions. In a previous study (*N* = 20) (16), the inter-rater agreement of the single EAFI items ranged from 0.6 to 1.0 with an average kappa of 0.84 and Cronbach's alphas above 0.88. Self-disorders were assessed on a lifetime basis with the Examination of Anomalous Self-Experience (EASE) (12). The EASE is a checklist for a semi-structured interview and consists of 57 items divided into five domains: Consciousness, presence, corporality, demarcation and solipsism (see Supplementary Table 2, for an overview of the domains and items). The EASE has demonstrated high internal consistency and inter-rater reliability (23, 24). ARR was trained and certified as EASE-rater by Dr. Julie Nordgaard, an official instructor and director of the EASE-courses. Before the study commenced, kappa-reliability was assessed (*N* = 20) with an average kappa of 0.74. Finally, we constructed a scale targeting non-psychotic, subjective perceptual disturbances, derived from the Bonn Scale for the Assessment of Basic Symptoms (BSABS) in continuity with previous studies in our group (24, 37) (see Supplementary Table 3). For the EAFI, EASE and perceptual disturbance scale, we rated the presence or absence (not severity or duration) of the items. Absent or questionably present items were scored 0, whereas definitely present items were scored 1. For the analysis, the main items were explored dimensionally (summing up the main items rated as present). As in previous EASE-studies (11) we did not additionally count the subtypes. Experiences associated with substance-abuse were not rated.

We retrospectively assessed the onset of anomalies of imagination asking the patients for their best estimate of the age when they first had such experiences. We categorized their responses into childhood (<12 years), adolescence (13–17 years) and adulthood (>18 years).

The IQ was assessed in the patient-groups by a computerized test, Intelligenz-Struktur-Test 2000R (54), and comprised verbal-, numerical-, and figurative-spatial-IQ represented by four selected subtests: analogies, sentence completion, sequences of numbers, and matrices. The results of these subtests were summed into a global IQ score used for data-analyses.

The assessment of the control group of students included, apart from the EAFI, a psychosocial history, a description of illness evolution, if such were elicited, and the examination of lifetime general psychopathology described above (OPCRIT, additional items from SADS-L and expressive features), but did not include the EASE, BSABS or PANSS.

### Allocation of Research Diagnoses

The research diagnoses were allocated according to DSM-5 and ICD-10. These were made as best-estimate consensus between the interviewer (AR) and the last author (JP) after a meeting assessing all relevant material from the assessment (interview summary, videos, ratings of instruments and information from hospital charts, which also contained second informant descriptions). For the purpose of analysis, the DSM-5 diagnoses were imposed the following hierarchy consistent with our previous studies: (1) schizophrenia, (2) other non-affective and non-organic psychosis, (3) bipolar disorder, (4) major depression, (5) schizotypal personality disorder, (6) OCD and related disorders, (7) other diagnosis (e.g., anxiety disorders, ADHD, and personality disorders other than schizotypal). The high priority given to schizotypal personality disorder reflects the study's focus on schizophrenia-spectrum disorders. OCD was also emphasized in the hierarchy due to the relevance of obsessions and obsessive-like symptomatology to the concept of anomalies of imagination.

### Statistical Analysis

In the analyses we used DSM-5 diagnoses comparing the following four groups (*N* = 81): (1) schizophrenia and other non-affective psychosis (jointly called “non-affective psychosis”) (*N* = 32), (2) schizotypal personality disorder (*N* = 15), (3) other mental illness (*N* = 16), and (4) a control group of healthy controls (*N* = 18). The diagnostic groups served as independent variable whereas the psychopathological scales (EAFI, EASE, perceptual disturbances, PANSS positive, negative, and general subscales) and sociodemographic variables (age, GAF-F) constituted dependent variables explored by parametric Analysis of variance (ANOVA) and non-parametric Kruskal-Wallis test. For *post hoc* pairwise comparisons of the groups we used t-test and Mann-Whitney U-test. We tested if the residuals from the models where normally distributed with Shapiro-Wilks test. χ^2^-test was used to examine if the distribution of subjects could be assumed independent between two categorical variables. Spearman correlations were calculated between EAFI score and the continuous variables (IQ, GAF-F, years since first symptom). We used Stata/SE 14.1 (StataCorp LP, College Station, Texas; www.stata.com) and SPSS version 22. The significance level was 0.05.

The network analysis, adopted to define the interrelations among psychopathological dimensions, was applied to the whole clinical sample (*N* = 63) in order to achieve sufficient power. The associations of EAFI with EASE, the perceptual disturbance scale derived from BSABS and the three dimensions of the PANSS (positive, negative and general symptoms) were tested by Pearson's correlation coefficient. When the sample size is sufficient, parametric tests are robust enough to withstand violations of the normal distribution (55). Correlations between variables were interpreted in term of effect size, with thresholds of 0.20 (small); 0.50 (moderate); and 0.80 (large). The network plot of the correlation data frame was generated through the “corrr” package running in R (56). In the graphic plot the variables that are more highly correlated appear closer together and are joined by stronger paths. The proximity of the points was determined using multidimensional clustering (57). Comparisons between correlations were calculated with the test of Steiger with the EAFI as referent term by using the routine accessible at http://quantpsy.org/corrtest/corrtest2.htm (58). The significance level was 0.05. In network analyses addressing psychopathology, multiple testing correction is usually not feasible (59). Here, we investigate a specific research hypothesis and expect to find a pattern of relations in the network plot consistent with that hypothesis.

Since EAFI and EASE share a limited fraction of partly overlapping items, the analyses were repeated removing these items from the EASE domains (EASE items 1.1, 1.2, 1.3, 1.6, 1.8, 1.10, and 2.2.1).

## Results

The sample characteristics are presented in Table 1. No significant associations were observed between EAFI score and age, gender, duration of illness or IQ. All patients scored above 70 on the IQ test. Among patients, EAFI score correlated negatively with social and occupational level of functioning (Spearman's ρ = −0.539, *P* < 0.0001, *N* = 63) assessed with the GAF-functioning subscale.

**Table 1:** Sociodemographic characteristics of the sample and distribution of psychopathology in the groups.

	**NAP**		**SPD**		**OMI**		**Students**		
	**Mean (SD)**	**Range**	**Mean (SD)**	**Range**	**Mean (SD)**	**Range**	**Mean (SD)**	**Range**	**Test-statistic (P)**
N	32		15		16		18		
Gender, F/M	21/11		10/5		13/3		14/4		χ^2^(3) = 1.81 (*P* = 0.612)
Age, years	30.1 (6.8)	19–42	27.3 (5.8)	18–37	32 (7.2)	20–42	27.5 (9.2)	19–53	*H*(3) = 6.54 (*P* = 0.088)
Years since first symptom	10.8 (7.2)	1–25	7.3 (4.8)	2–19	8.3 (6.8)	0.2–20			*H*(2) = 3.12 (*P* = 0.212)
GAF-F^a^	37.7 (9.9)	25–61	48.3 (14.4)	35–75	62.7 (12.9)	45–85	85.7 (7.6)	71–95	*F* _(2, 60)_ = 76.34 (*P* < 0.0001)
EAFI^b^	8.7 (2.6)	2–13	7.9 (1.6)	5–10	2.8 (1.8)	1–6	0.4 (0.9)	0–3	*H*(3) = 59.48 (*P* < 0.001)
EASE^c^	18.9 (7.1)	2–33	15.4 (3.8)	9–22	5.4 (3.1)	2–10			*F*_(2, 60)_ = 30.95 (*P* < 0.0001)
Perceptual disturbances^d^	2.9 (2.1)	0–7	1.7 (1.6)	0–5	1.0 (1.3)	0–3			*H*(3) = 11.70 (*P* < 0.003)
PANSS-pos^e^	19.3 (4.2)	12–30	13.8 (3.2)	10–21	8.7 (1.9)	7–13			*F*_(2, 60)_ = 49.58 (*P* < 0.0001)
PANSS-neg^e^	17.9 (6.6)	7–30	12.9 (5.3)	7–25	7.4 (1.0)	7–11			*F*_(2, 60)_ =20.41 (*P* < 0.0001)
PANSS-general^e^	31.6 (6.7)	15–42	23.7 (5.4)	13–32	15.9 (3.8)	10–24			*F*_(2, 60)_ = 40.35 (*P* < 0.0001)

*NAP, schizophrenia and non-affective psychosis; SPD, schizotypal personality disorder; OMI, other mental illness. Cronbach's alpha = 0.872 for EAFI.*

*H-test statistic and p-value are from the non-parametric Kruskal-Wallis test for equality of diagnostic groups with post hoc Mann-Whitney U-test. F-test statistic and p-value are from parametric ANOVA test for equality of diagnostic groups with post hoc t-test.*

*Post-hoc tests:*

a *NAP < SPD < OMI < Students;*

b *NAP = SPD > OMI > students;*

c *NAP = SPD > OMI;*

d *NAP > SPD = OMI;*

e *NAP > SPD > OMI. Significance level 0.05*.

Anomalies of imagination aggregated significantly within the schizophrenia spectrum as compared to patients outside the spectrum and healthy controls (Table 1). There was no significant difference in EAFI score between schizophrenia/non-affective psychosis and schizotypal disorder (*P* = 0.16, Mann-Whitney U-test). The group with other mental illness [major depression (*N* = 4) and OCD (*N* = 12)] scored substantially lower than the schizophrenia-spectrum patients (*P* < 0.001, Mann-Whitney U-test) but significantly higher than the group of healthy controls (*P* < 0.001, Mann-Whitney U-test).

The patients retrospectively reported that the anomalies of imagination were experienced from adolescence or childhood (<18 years) in 79% of cases in the group with schizophrenia or non-affective psychosis and 85% of cases in the group with schizotypal disorder.

**Figure 1:**
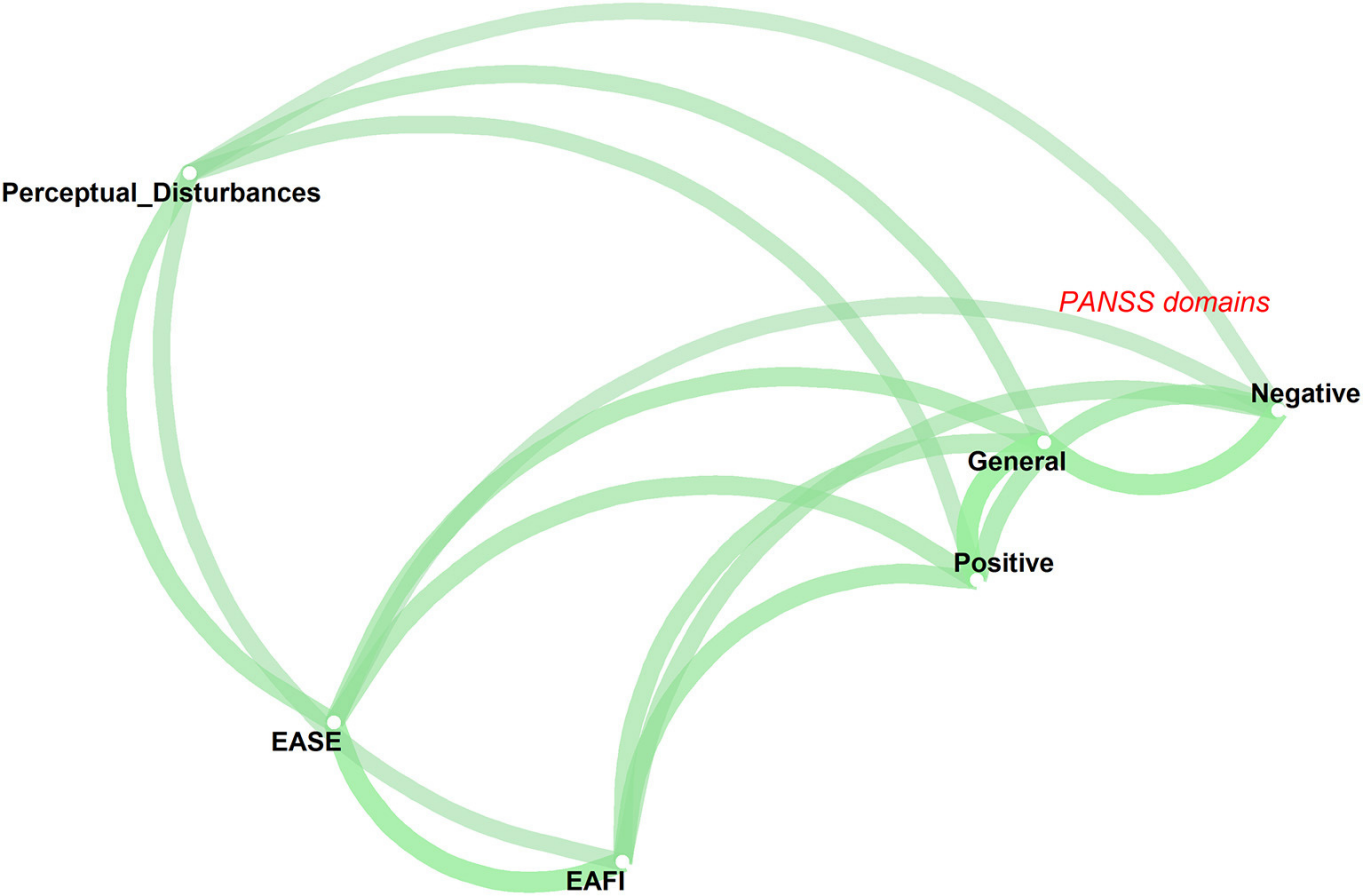
Psychopathological network showing the pairwise associations among anomalies of imagination (EAFI), self-disorders (EASE), perceptual disturbance and positive, negative and general symptoms (PANSS dimensions). The thickness of the lines connecting the variables is proportional to their correlations with the target variable.

Correlational analysis and their differential significance (Steiger's test) in the clinical sample (*N* = 63) are presented in Tables 2, 3. Briefly, anomalies of imagination (EAFI) had moderate to large effect size correlations with self-disorders (EASE) and positive, general and negative state symptomatology (PANSS domains) (numbered in declining order of magnitude) and relatively weaker effect size correlation with perceptual disturbance (Table 2). Overall, anomalies of imagination, self-disorders and the positive, negative and general symptom domains of the PANSS had moderate to large effect size correlations with each other, while their effect size correlations with perceptual disturbance were, on average, relatively weaker. Self disorders were significantly stronger correlated with anomalies of imagination than with positive, negative and general state symptomatology as well as perceptual disturbances (Table 3). Positive, negative and general symptoms were significantly stronger inter-correlated, apart from the “positive-negative symptoms” association, compared with their correlation with the EAFI. Overall, the multidimensional clustering algorithm plotted anomalies of imagination in the same cluster with self-disorders, separating them from the PANSS dimensions as well as perceptual disturbance (Figure 1). These results remained unchanged when we used the correlations among variables after removing the partly overlapping items between EASE and EAFI.

**Table 2:** Correlations among psychopathological variables.

				**PANSS**		
	**EAFI**	**EASE**	**Perceptual disturbances**	**Positive**	**Negative**	**General**
EAFI	–					
EASE	0.763^**^	–				
Percept. Dist.	0.479^**^	0.601^**^	–			
Positive	0.650^**^	0.595^**^	0.478^**^	–		
Negative	0.549^**^	0.434^**^	0.405^*^	0.645^**^	–	
General	0.572^**^	0.605^**^	0.495^**^	0.816^**^	0.760^**^	–

*Pearson's correlation.*

* *p < 0.001.*

** *p < 0.0001. N = 63*.

**Table 3:** Comparison of correlations.

			**PANSS**		
	**EASE**	**Perceptual disturbances**	**Positive**	**Negative**	**General**
EASE	–	***z*** **= −1.95**, ***p*** **= 0.05**	***z*** **= −2.36**, ***p*** **= 0.02**	***z*** **= −3.83**, ***p*** **< 0.0001**	***z*** **= −2.06**, ***p*** **= 0.04**
Percept. Dist.		**–**	*z* = −0.01, *p* = 0.99	*z* = −0.69, *p* = 0.49	*z* = 0.16, *p* = 0.87
Positive			**–**	*z* = −0.06, *p* = 0.95	***z*** **= 2.43**, ***p*** **= 0.01**
Negative				**–**	***z*** **= 2.64**, ***p*** **= 0.01**
General					**–**

*N = 63. All comparisons were assessed with the Steiger's test, by using the EAFI as referent term (two-tailed test). Statistically significant results (p < 0.05) are in bold.*

*The cross-section cell between a column and a row tests for a significant difference between the correlation of the column item with the row-item and the correlation of the column-item with the EAFI, adjusting for the correlation of the row item with the EAFI*.

## Discussion

We found that anomalies of imagination, explored by the EAFI, aggregated substantially and significantly within the schizophrenia spectrum compared to other mental illness and healthy controls. The group with other mental illness had a low life-time occurrence of anomalies of imagination but scored significantly higher than healthy controls, who rarely reported these disturbances. Furthermore, network analysis showed that anomalies of imagination were closely associated with disorders of basic self relative to their mutual correlation with perceptual disturbances and positive, negative and general state psychopathology. Whereas, we have previously published clinical vignettes and data regarding reliability of the EAFI (1, 2, 16, 17), this is the first systematic, empirical study of anomalies of imagination.

In our view, the anomalies of imagination do not simply reflect a high score on a putative general dimension of vividness regarding mental imagery (5, 6) but rather indicate a more specific disturbance of subjective experience. More specifically, the relatively close inter-connectedness between anomalies of imagination and self-disorders may reflect that the anomalies of imagination and minimal self share a common core structure rather than their co-existing as two independent clusters of symptoms (2). That is, they are closely interrelated facets of a central disturbance of the basic structure of subjectivity. From a phenomenological-conceptual perspective, indeed, anomalies of imagination articulate the same fundamental disorder of ipseity (the first-person perspective) as self-disorders. Ipseity involves existing as a subject of experience that is at one with itself (self-coinciding) at any given moment (19, 60). Like all experience, ordinary imaginative and introspective activity is saturated with a first-personal character (“mineness,” “for-me-ness”) and there is no sense of experiential distance between the experiencing subject and thoughts, images, emotions etc. [i.e., introspective consciousness is not experienced as an “interior” container or “theater of consciousness” (8, 9)]. Anomalies of imagination seem to manifest a disturbance of this normally incessant, coherent articulation of the first-person perspective. Patients describe the appearance of “images,” “inner movies,” “scenarios” etc. that appear disconnected from their main line of thinking. Imaginations acquire an autonomous flow of content, which to the patient appears independent from his or her intentions or motivations, therefore substantiating a contraction of experienced “mineness” and agency of mental life. This anonymization of the experiential field is typically intertwined with the appearance of an experiential distance, where the person experiences a sense of observing the “inner” life rather than experiencing imagery as permeated by subjectivity “from within.” The spatialization of images reflects an even more pronounced distortion of the introspective field of awareness. Imagery are here experienced as thing-like, autonomous entities further manifesting a diminished or unstable first-personal character of the experience.

Although preliminary, our findings suggest a relative specificity of anomalies of imagination to schizophrenia spectrum conditions and highlight their potential psychopathological significance. The results seem to indicate that anomalies of imagination are not a consequence of psychotic state symptomatology since they are equally present in schizotypal disorder and cluster separately in the network analysis. Interestingly, the far majority of schizophrenia-spectrum patients retrospectively estimated the onset of these disturbances to childhood or adolescence. Apart from facilitating a certain awareness and understanding of patients' experience, crucial to therapy and general clinical work, the anomalies of imagination, have a potential relevance for differential diagnosis and early detection. Especially, in patients presenting with diagnostically unspecific complaints related to the modality of imagination such as obsessions, ruminations and various disturbing ideations (25).

The study has important limitations: (1) The sample is relatively small, although this must be seen in the light of a very thorough and time-consuming psychopathological assessment, (2) the groups are heterogenous in terms of illness stage and, in regard of the group with of other mental illness, diagnostic status, and (3) the clinical sample was biased toward females, probably because the inclusion criteria and places of recruitment naturally tended to exclude patients with agitation, substance abuse or legal status. Finally, the network analysis was carried out in a cross-diagnostic sample in order to achieve sufficient power.

In sum, the study suggests that the anomalies of imagination, explored with the EAFI, are highly characteristic of schizophrenia-spectrum disorder and have a close empirical relation with self-disorders. Together with phenomenological-conceptual considerations, the findings support that anomalies of imagination reflect a structural disorder of subjectivity articulated by the notion of disturbed ipseity (first-person perspective). Future studies should address the temporal stability of anomalies of imagination and their longitudinal intercorrelations with self-disorders and other schizophrenia-spectrum psychopathology. The relation of anomalies of imagination to disturbances of intersubjectivity should be further explored (61) as well as possible transitions of anomalies of imagination into certain psychotic, hallucinatory-like experiences. Moreover, studies are needed regarding their developmental origin and potential value as risk markers of transition to schizophrenia-spectrum disorders.

## Data Availability Statement

The datasets presented in this article are not readily available because it contains sensitive personal information. Requests to access the datasets should be directed to arr@dadlnet.dk.

## Ethics Statement

Ethical review and approval was not required for the study on human participants in accordance with the local legislation and institutional requirements. The patients/participants provided their written informed consent to participate in this study. Written informed consent was obtained from the individual(s) for the publication of any potentially identifiable images or data included in this article.

## Author Contributions

ARR, JP, and AR contributed to the study conception and design. Material preparation and data collection were performed by ARR. Analysis was performed by DS, AP, ARR, and AR. The first draft of the manuscript was written by ARR and all authors commented on subsequent versions of the manuscript, read, and approved the final manuscript.

## Funding

This work was supported by The Faculty of Health Sciences, University of Copenhagen (grant to ARR).

## Conflict of Interest

The authors declare that the research was conducted in the absence of any commercial or financial relationships that could be construed as a potential conflict of interest.

## Publisher's Note

All claims expressed in this article are solely those of the authors and do not necessarily represent those of their affiliated organizations, or those of the publisher, the editors and the reviewers. Any product that may be evaluated in this article, or claim that may be made by its manufacturer, is not guaranteed or endorsed by the publisher.

## References

[1] Rasmussen AR Parnas J. Pathologies of imagination in schizophrenia spectrum disorders. Acta Psychiatr Scand. (2015) 131:157–61. 10.1111/acps.12323 25098736

[2] Rasmussen AR Parnas J. Anomalies of imagination and disordered self in schizophrenia spectrum disorders. Psychopathology. (2015) 48:317–23. 10.1159/000431291 26352692

[3] Pearson DG Deeprose C Wallace-Hadrill SM Burnett Heyes S Holmes EA. Assessing mental imagery in clinical psychology: a review of imagery measures and a guiding framework. Clin Psychol Rev. (2013) 33:1–23. 10.1016/j.cpr.2012.09.001 23123567 PMC3545187

[4] Kosslyn SM Thompson WL Ganis G. The Case for Mental Imagery. New York, NY: Oxford University Press (2006). p. 248.

[5] Sack AT van de Ven VG Etschenberg S Schatz D Linden DE. Enhanced vividness of mental imagery as a trait marker of schizophrenia? Schizophr Bull. (2005) 31:97–104. 10.1093/schbul/sbi011 15888429

[6] Oertel V Rotarska-Jagiela A van de Ven V Haenschel C Grube M Stangier U. Mental imagery vividness as a trait marker across the schizophrenia spectrum. Psychiatry Res. (2009) 167:1–11. 10.1016/j.psychres.2007.12.008 19345421

[7] Bell V Halligan PW. Additional data on whether vividness of visual mental imagery is linked to schizotypal traits in a non-clinical population. Psychiatry Res. (2010) 178:568–9. 10.1016/j.psychres.2009.05.003 20537724

[8] Sartre J-P. The Imaginary: A Phenomenological Psychology of the Imagination. London: New York, NY: Routledge (2010).

[9] Husserl E. Phantasy, Image Consciousness, and Memory, 1898–1925. Dordrecht: Springer (2005).

[10] Thompson E. Look again: phenomenology and mental imagery. Phenomenol Cogn Sci. (2007) 6:137–70. 10.1007/s11097-006-9031-127005586

[11] Nordgaard J Henriksen MG Jansson L Handest P Moller P Rasmussen AR. Disordered selfhood in schizophrenia and the examination of anomalous self-experience: accumulated evidence and experience. Psychopathology. (2021) 54:275–81. 10.1159/000517672 34384082 PMC8686724

[12] Parnas J Moller P Kircher T Thalbitzer J Jansson L Handest P. EASE: examination of anomalous self-experience. Psychopathology. (2005) 38:236–58. 10.1159/000088441 16179811

[13] Gross G Huber G Klosterkötter J Lintz M. Bonner Skala Für die Beurteilung von Basissymptomen. Berlin: Springer Verlag (1987).

[14] Schultze-Lutter S Addington J Rurhmann S. Schizophrenia Proneness Instrument, Adult Version (SPI-A). Rome: Giovanni Fioriti Editore (2007).

[15] Sass L Pienkos E Skodlar B Stanghellini G Fuchs T Parnas J. EAWE: Examination of Anomalous World Experience. Psychopathology. (2017) 50:10–54. 10.1159/000454928 28268224

[16] Rasmussen AR Stephensen H Nordgaard J Parnas J. A Phenomenological approach to psychopathology of imagination: development of a descriptive instrument–examination of anomalous fantasy and imagination. Psychopathology. (2018) 51:210–5. 10.1159/000488463 29758557

[17] Rasmussen AR Stephensen H Parnas J. EAFI Examination of anomalous fantasy and imagination. Psychopathology. (2018) 51:216–26. 10.1159/000488464 29758549 PMC6106139

[18] Henriksen MG Raballo A Nordgaard J. Self-Disorders and psychopathology: a systematic review. Lancet Psychiatry. (2021) 8:1001–12. 10.1016/S2215-0366(21)00097-3 34688345

[19] Zahavi D. Subjectivity and Selfhood: Investigating the First-Person Perspective. Cambridge, MA: MIT Press (2005).

[20] Sass LA Parnas J. Schizophrenia, consciousness, and the self. Schizophr Bull. (2003) 29:427–44. 10.1093/oxfordjournals.schbul.a007017 14609238

[21] Parnas J Henriksen MG. Disordered self in the schizophrenia spectrum: a clinical and research perspective. Harv Rev Psychiatry. (2014) 22:251–65. 10.1097/HRP.0000000000000040 25126763 PMC4219858

[22] Parnas J Handest P. Phenomenology of anomalous self-experience in early schizophrenia. Compr Psychiatry. (2003) 44:121–34. 10.1053/comp.2003.50017 12658621

[23] Moller P Haug E Raballo A Parnas J Melle I. Examination of anomalous self-experience in first-episode psychosis: interrater reliability. Psychopathology. (2011) 44:386–90. 10.1159/000325173 21847006

[24] Nordgaard J Parnas J. Self-disorders and the schizophrenia spectrum: a study of 100 first hospital admissions. Schizophr Bull. (2014) 40:1300–7. 10.1093/schbul/sbt239 24476579 PMC4193705

[25] Rasmussen AR Nordgaard J Parnas J. Schizophrenia-spectrum psychopathology in obsessive-compulsive disorder: an empirical study. Eur Arch Psychiatry Clin Neurosci. (2020) 270:993–1002. 10.1007/s00406-019-01022-z 31129700 PMC7599137

[26] Zandersen M Parnas J. Exploring schizophrenia spectrum psychopathology in borderline personality disorder. Eur Arch Psychiatry Clin Neurosci. (2019). 10.1007/s00406-019-01039-4 31289925 PMC7599140

[27] Nilsson M Arnfred S Carlsson J Nylander L Pedersen L Mortensen EL. Self-disorders in asperger syndrome compared to schizotypal disorder: a clinical study. Schizophr Bull. (2019) 46:121–9. 10.1093/schbul/sbz036 31050761 PMC6942161

[28] Haug E Lien L Raballo A Bratlien U Oie M Andreassen OA. Selective aggregation of self-disorders in first-treatment DSM-IV schizophrenia spectrum disorders. J Nerv Ment Dis. (2012) 200:632–6. 10.1097/NMD.0b013e31825bfd6f 22759943

[29] Raballo A Monducci E Ferrara M Fiori Nastro P Dario C. group R. Developmental vulnerability to psychosis: Selective aggregation of basic self-disturbance in early onset schizophrenia. Schizophr Res. (2018) 201:367–72. 10.1016/j.schres.2018.05.012 29804931

[30] Raballo A Parnas J. The silent side of the spectrum: schizotypy and the schizotaxic self. Schizophr Bull. (2011) 37:1017–26. 10.1093/schbul/sbq008 20176859 PMC3160219

[31] Raballo A Saebye D Parnas J. Looking at the schizophrenia spectrum through the prism of self-disorders: an empirical study. Schizophr Bull. (2011) 37:344–51. 10.1093/schbul/sbp056 19528205 PMC3044618

[32] Raballo A Poletti M Preti A Parnas J. The self in the spectrum: a meta-analysis of the evidence linking basic self-disorders and schizophrenia. Schizophr Bull. (2021) 47:1007–17. 10.1093/schbul/sbaa201 33479736 PMC8266610

[33] Nelson B Thompson A Yung AR. Basic self-disturbance predicts psychosis onset in the ultra high risk for psychosis “prodromal” population. Schizophr Bull. (2012) 38:1277–87. 10.1093/schbul/sbs007 22349924 PMC3494062

[34] Koren D Tzivoni Y Schalit L Adres M Reznik N Apter A. Basic self-disorders in adolescence predict schizophrenia spectrum disorders in young adulthood: a 7-year follow-up study among non-psychotic help-seeking adolescents. Schizophr Res. (2019) 216:97–103. 10.1016/j.schres.2019.12.022 31889574

[35] Raballo A Preti A. Temporal stability of self-disorders and longitudinal unfolding of symptom dimensions: a complementary analysis. Schizophr Res. (2018) 195:78–9. 10.1016/j.schres.2017.08.024 28844433

[36] Nordgaard J Handest P Vollmer-Larsen A Saebye D Pedersen JT Parnas J. Temporal persistence of anomalous self-experience: a 5 years follow-up. Schizophr Res. (2016) 179:36–40. 10.1016/j.schres.2016.10.001 27720316

[37] Nordgaard J Nilsson LS Saebye D Parnas J. Self-disorders in schizophrenia-spectrum disorders: a 5-year follow-up study. Eur Arch Psychiatry Clin Neurosci. (2017) 268:713–8. 10.1007/s00406-017-0837-3 28865064 PMC6132940

[38] Raballo A Preti A. The self in the spectrum: a closer look at the temporal stability of self-disorders in schizophrenia. Psychopathology. (2018) 51:285–9. 10.1159/000488645 29734188

[39] Rasmussen AR Saebye D Parnas J. Anomalies of imagination in the schizophrenia-spectrum: Empirical findings. Schizophr Res. (2019) 206:458–9. 10.1016/j.schres.2018.11.019 30503368

[40] Parnas J Cannon TD Jacobsen B Schulsinger H Schulsinger F Mednick SA. Lifetime DSM-III-R diagnostic outcomes in the offspring of schizophrenic mothers. Results from the copenhagen high-risk study. Arch Gen Psychiatry. (1993) 50:707–14. 10.1001/archpsyc.1993.01820210041005 8357296

[41] Parnas J Licht D Bovet P. Cluster A personality disorders: a review. In: Maj M, Akiskal HS, Mezzich JE, Okasha A, editors. Personality Disorders, Volume 8. Chichester: John Wiley and Sons, Ltd. (2005), 1–124.

[42] Pedersen CB Mors O Bertelsen A Waltoft BL Agerbo E McGrath JJ. A comprehensive nationwide study of the incidence rate and lifetime risk for treated mental disorders. JAMA Psychiatry. (2014) 71:573–81. 10.1001/jamapsychiatry.2014.16 24806211

[43] Jaspers K. General Psychopathology. Chicago, IL: University of Chicago Press (1963).

[44] Nordgaard J Sass LA Parnas J. The psychiatric interview: validity, structure, and subjectivity. Eur Arch Psychiatry Clin Neurosci. (2013) 263:353–64. 10.1007/s00406-012-0366-z 23001456 PMC3668119

[45] Jansson L Nordgaard J. The Psychiatric Interview for Differential Diagnosis. Cham: Springer (2016).

[46] Parnas J Handest P Jansson L Saebye D. Anomalous subjective experience among first-admitted schizophrenia spectrum patients: empirical investigation. Psychopathology. (2005) 38:259–67. 10.1159/000088442 16179812

[47] Parnas J Raballo A Handest P Jansson L Vollmer-Larsen A Saebye D. Self-experience in the early phases of schizophrenia: 5-year follow-up of the Copenhagen Prodromal Study. World Psychiatry. (2011) 10:200–4. 10.1002/j.2051-5545.2011.tb00057.x 21991279 PMC3188774

[48] McGuffin P Farmer A Harvey I. A polydiagnostic application of operational criteria in studies of psychotic illness. Development and reliability of the OPCRIT system. Arch Gen Psychiatry. (1991) 48:764–70. 10.1001/archpsyc.1991.01810320088015 1883262

[49] Wing JK. Present State Examination. 9th ed. Cambridge: Cambridge University Press (1974).

[50] Endicott J Spitzer RL. A diagnostic interview: the schedule for affective disorders and schizophrenia. Arch Gen Psychiatry. (1978) 35:837–44. 10.1001/archpsyc.1978.01770310043002 678037

[51] Vaever MS Licht DM Moller L Perlt D Jorgensen A Handest P. Thinking within the spectrum: schizophrenic thought disorder in six Danish pedigrees. Schizophr Res. (2005) 72:137–49. 10.1016/j.schres.2004.04.001 15560959

[52] Kay SR Fiszbein A Opler LA. The positive and negative syndrome scale (PANSS) for schizophrenia. Schizophr Bull. (1987) 13:261–76. 10.1093/schbul/13.2.261 3616518

[53] Endicott J Spitzer RL Fleiss JL Cohen J. The global assessment scale. A procedure for measuring overall severity of psychiatric disturbance. Arch Gen Psychiatry. (1976) 33:766–71. 10.1001/archpsyc.1976.01770060086012 938196

[54] Liepmann D. Intelligenz-Struktur-Test 2000 R (I-S-T 2000 R). 2. erweiterte und überarbeitete Auflage ed. Göttingen: Hogrefe (2007).

[55] Altman DG. Practical Statistics for Medical Research. https://www.google.com/search?sxsrf=AOaemvKHskasha0dB0fGujQg4RaBrJ7pLA:1640419731011&q=London&stick=H4sIAAAAAAAAAOPgE-LUz9U3SDE3LjNW4gAxTbIKcrSMMsqt9JPzc3JSk0sy8_P084vSE_MyqxJBnGKrjNTElMLSxKKS1KJihZz8ZLDwIlY2n_y8lPy8HayMACyemoZXAAAA&sa=X&ved=2ahUKEwi6reXav_70AhX_6XMBHYN0Dv4QmxMoAXoECCAQAwLondon:ChapmanandHall (1991).

[56] Jackson S. Corrr: Correlations in R—R Package. Version 0.3.0. Available online at: https://CRAN.R-project.org/package=corrr (accessed January 3, 2022).

[57] Jackson MO. The past and future of network analysis in economics. In: Bramoullé Y, Galeotti A, Rogers B, editors. Oxford Handbook on the Economics of Networks. New York, NY: Oxford University Press (2016). p. 71–81.

[58] Lee IA Preacher KJ. Calculation for the Test of the Difference Between Two Dependent Correlations With One Variable in Common [Computer Software]. (2013). Available online at: http://quantpsy.org/ (accessed October 1, 2021).

[59] Epskamp S Borsboom D Fried EI. Estimating psychological networks and their accuracy: a tutorial paper. Behav Res Methods. (2018) 50:195–212. 10.3758/s13428-017-0862-1 28342071 PMC5809547

[60] Parnas J Henriksen MG. Mysticism and schizophrenia: a phenomenological exploration of the structure of consciousness in the schizophrenia spectrum disorders. Conscious Cogn. (2016) 43:75–88. 10.1016/j.concog.2016.05.010 27258928

[61] Goze T Fazakas I. Imagination and self disorders in schizophrenia: a review. Psychopathology. (2020) 53:1–10. 10.1159/000509488 33059352

