# Supplementary material for: Anomalies of Imagination, Self-Disorders, and Schizophrenia Spectrum Psychopathology: A Network Analysis
Source: Front Psychiatry. 2022 Jan 17;12:808009. doi: 10.3389/fpsyt.2021.808009 (PMC8801416; doi:10.3389/fpsyt.2021.808009)
Supplement: Supplementary file 1 [file Data_Sheet_1.doc]

**Supplementary Material**

**Anomalies of imagination, self-disorders and schizophrenia spectrum psychopathology: a network analysis**

Andreas Rosén Rasmussen, Andrea Raballo, Antonio Preti, Ditte Sæbye, Josef Parnas

Correspondence:

Andreas Rosén Rasmussen, MD

Mental Health Center Amager, University of Copenhagen,

Digevej 110, 2300 Kbh. S, Denmark

E-mail: [Arr@dadlnet.dk](mailto:Arr@dadlnet.dk)

| **Supplementary material. Table 1.** Overview of the main items of the Examination of Anomalous Fantasy and Imagination (EAFI) with brief descriptions. | | | |  |
| --- | --- | --- | --- | --- |
| 1 | Spatio-temporal constancy | A fixed quasi-spatial structure of the image that endures as a temporally, stable object. The patient can describe fixed locations and relations between the elements of the imagery. Or the image is liable to inspection and exploration like a stable, perceived object such as a picture on a wall. | |  |
| 2 | Autonomy | The imagery acquires its own flow, sequence or development, ‘like a movie’, independent from the willed intention of the subject. | |  |
| 3 | Localization and sense of experiential distance | The imagery is experienced as localized to a specific part of the head or brain. Or there is an experiential distance between the sense of the image and the sense of self (observing one’s imaginations ‘from afar’ as an inner object). | |  |
| 4 | Intense affects |  | |  |
| 5 | Imagination as multimodal perceptualization | The perceptualized imagery is accompanied by as-if experiences resembling other perceptual modalities such as an acoustic or tactile quality. | |  |
| 6 | Anomalous vivid imaginary experience, unspecified | Certain border phenomena to item 1-5. | |  |
| 7 | Violent, macabre or bizarre content |  | |  |
| 8 | Ruminations-obsessions | Includes true obsessions, pseudo-obsessions, primary ruminations, secondary ruminations. | |  |
| 9 | Disturbance of control | A lack of ability to repress unwanted imaginative experiences (at least temporarily) by an effort of the will. | |  |
| 10 | Loss of ipseity | The imagery appears foreign, lacking ‘mineness’. ‘As if it was not my own thoughts’ (that is, not with psychotic quality). | |  |
| 11 | Image interference | Imagery appears suddenly, disconnected thematically from the main line of thinking. | |  |
| 12 | Image pressure | Parallel to thought pressure. | |  |
| 13 | Existential orientation in fantasy | The themes and concerns of phantasy life appear to express disturbances of the basic structure of being an experiential subject embedded in the world. | |  |
| 14 | Preoccupation with fantasy life | Excessive amount of time and energy spent phantasizing or daydreaming. Often with idiosyncratic existential importance ascribed to certain recurrent phantasies | |  |
| 15 | Disturbances of irreality | Includes several subtypes ranging from an insecurity about the modality (‘was this a memory or a phantasy’?) to micro-psychotic episodes where the distinction between an imaginary experience and the shared, surrounding, perceptual world is briefly lost (seconds to a few minutes). | |  |
| 16 | Disturbance of insight | The content of obsessive phenomena (item 8), violent or idiosyncratic imaginations (item 7 and 13) is not considered absurd, excessive or irrelevant by the subject. Includes short-lasting lack of insight, only *during* the imaginative experience. | |  |
| For complete definitions, further descriptions and examples of the items, please see: Rasmussen AR, Stephensen H, Parnas J. EAFI: Examination of Anomalous Fantasy and Imagination. *Psychopathology* 2018;51:216-226  Cronbach’s alpha of EAFI in this study was 0.872 (N=81) and 0.788 (N=63) | | | |  |
| **Supplementary material. Table 2.** Overview of the domains and main items of the Examination of Anomalous Self-Experience (EASE) | | | | |
| **Consciousness** (Domain 1; ‘Cognition and stream of consciousness’) | | | | |
| 1.1 Thought interference  1.2 Loss of thought ipseity  1.3 Thought pressure  1.4 Thought block  1.5 Silent thought echo  1.6 Ruminations-obsessions  1.7 Perceptualization of inner speech or thought  1.8 Spatialization of experience  1.9 Ambivalence | | | 1.10 Inability to discriminate modalities of intentionality  1.11 Disturbance of thought initiative/intentionality  1.12 Attentional disturbances  1.13 Disorder of short-term memory  1.14 Disturbance of time experience  1.15 Discontinuous awareness of own action  1.16 Discordance between intended expression and the expressed  1.17 Disturbance of expressive language function | |
| **Presence** (Domain 2; ‘Self-awareness and presence’) | | | | |
| 2.1 Diminished sense of basic self  2.2 Distorted first-person perspective  2.3 Psychic depersonalization (self-alienation)  2.4 Diminished presence  2.5 Derealization  2.6 Hyperreflectivity (increased reflectivity)  2.7 I-split (‘‘Ich-Spaltung’’)  2.8 Dissociative depersonalization  2.9 Identity confusion | | | 2.10 Sense of change in relation to chronological age  2.11 Sense of change in relation to gender  2.12 Loss of common sense, lack of natural evidence  2.13 Anxiety  2.14 Ontological anxiety  2.15 Diminished transparency of consciousness  2.16 Diminished initiative  2.17 Hypohedonia  2.18 Diminished vitality | |
| **Corporality** (Domain 3; ‘Bodily experiences’) | | | | |
| 3.1 Morphological change  3.2 Mirror-related phenomena  3.3 Somatic depersonalization (bodily estrangement)  3.4 Psychophysical misfit and psychophysical split  3.5 Bodily disintegration | | | 3.6 Spatialization (objectification) of bodily experiences  3.7 Cenesthetic experiences  3.8 Motor disturbances  3.9 Mimetic experience (resonance between own movement and others’ movements) | |
| **Demarcation** (Domain 4; ‘Demarcation/Transitivism’) | | | | |
| 4.1 Confusion with the other  4.2 Confusion with one’s own specular image  4.3 Threatening bodily contact and feelings of fusion | | | 4.4 Passivity mood  4.5 Other transitivistic phenomena | |
| **Solipsism** (Domain 5; ‘Existential reorientation’) | | | | |
| 5.1 Primary self-reference phenomena  5.2 Feeling of centrality  5.3 Feeling as if the subject’s experiential field is the only extant reality  5.4 ‘‘As if ’’ feelings of extraordinary creative power or insight into hidden dimensions of reality | | | 5.5 ‘‘As if ’’ feeling that the experienced world is not truly real, as if it was only somehow apparent  5.6 Magical ideas linked to the subject’s way of experiencing  5.7 Existential or intellectual change  5.8 Solipsistic grandiosity | |
| For the complete definitions, further descriptions and examples of the items, please see: Parnas J, Moller P, Kircher T, et al. EASE: Examination of Anomalous Self-Experience. *Psychopathology* 2005; 38:236-258  Cronbach’s alpha of EASE in this study was 0.881 (N=63) | | | | |

| **Supplementary material. Table 3.** Perceptual disturbances |
| --- |
| Blurred vision  Partial vision  Transient blindness  Visual perceptual disturbances  Disruptions in the assessment of an object’s distance and size  Abnormally long persistent optical irritation  Hyperacusis  Changes in hearing  Abnormal sustained sound impression  Perceptual changes: olfactorial  Perceptual changes: taste  Disturbance in the perception of the importance of the observed |
| Overwhelming sensory input |
| Items derived from: Gross, G., Huber, G., Klosterkötter, J., Lintz, M. Bonner Skala Für die Beurteilung von Basissymptomen. Springer Verlag, 1987  Cronbach’s alpha of perceptual disturbance scale in this study was 0.641 (N=63) |
